# Editorial: Harnessing DNA Damage Response in Gynecologic Malignancies

**DOI:** 10.3389/fonc.2022.882925

**Published:** 2022-03-28

**Authors:** Ka Yu Tse, Jihong Liu, Sung-Jong Lee, David S. P. Tan

**Affiliations:** ^1^ Division of Gynaecological Oncology, Department of Obstetrics and Gynaecology, The University of Hong Kong, Hong Kong, Hong Kong SAR, China; ^2^ Department of Gynecologic Oncology, Sun Yat-sen University Cancer Centre, Guangzhou, China; ^3^ Department of Obstetrics & Gynecology. Seoul St. Mary’s Hospital, College of Medicine, The Catholic University of Korea, Seoul, South Korea; ^4^ Department of Haematology-Oncology, National University Cancer Institute, Singapore (NCIS) National University Hospital (NUH) Singapore, Singapore, Singapore

**Keywords:** DNA damage response, gynecologic malignancies, ovarian cancer, cervical cancer, homologous recombination repair deficiency

DNA damage and its related cellular process are one of the hallmarks of cancer (1). Yet cancer cells require DNA damage response (DDR) to maintain the genomic stability for cell survival. As many gynecological cancers have defects in the DDR and/or homologous recombination repair (HRR) pathways, this opens a therapeutic window. For example, poly(ADPribose) polymerase (PARP) inhibition impairs the repair of single-strand DNA breaks (SSB), accumulation of which results in double-strand breaks (DSB). Where homologous recombination repair deficiency (HRD) is present, such as cells harboring *BRCA1/2* pathogenic mutations, DSBs cannot be effectively repaired, and cell death follows. This Research Topic aims to provide an overview on latest advances related to DDR targeting in gynecologic malignancies.

Epithelial ovarian cancer (EOC) is a heterogeneous disease. High-grade serous carcinoma (HGSC) is the most common subtype which constitutes about 70% of all EOCs. Ovarian clear cell carcinoma (OCCC) is the second commonest, and its incidence is up to 20% in Asia. It is typically resistant to chemotherapy and is rarely associated with *BRCA1/2* mutations. Wong et al. reviewed the DDR status in OCCC, and showed that up to half of the patients had BRCAness phenotypes such as *BRCA1* hypermethylation, and about one-third of OCCC patients had mutation in HR genes especially *ATM.* Additionally, about half of OCCC also harbors loss-of-function mutations in *ARID1A*, while *PIK3CA* mutations are present in 20 – 46% of these tumors, and may still be exploited by DDR-targeted therapies including ATM/ATR inhibitors or mTOR pathway inhibitors as well as PARP inhibitors (PARPi).

PARP inhibitors are now a standard therapy for EOC patients who are sensitive to platinum-based chemotherapy and naïve to PARPi, and it is particularly effective in tumors with HRD. In the meta-analysis by Xu et al., which included over 1800 patients from five randomized controlled trials, olaparib, rucaparib and niraparib maintenance therapy led to prolonged progression-free survival (PFS) for patients with *BRCA* mutations as well as for the overall population in platinum-sensitive recurrent EOC compared with placebo. Rucaparib and niraparib were more often associated with grade 3 or 4 toxicity compared with olaparib. It is noteworthy that these trials were limited to patients with high-grade endometrioid or HGSC. Notably, each of these studies enrolled slightly different patient populations, and given the lack of any head to head comparisons on the efficacy and safety among different PARPi, it is still not possible to draw any conclusion to suggest which PARPi is the best.

Currently, the biomarkers used for selecting patients for PARPi are high-grade histology, platinum-sensitivity, *BRCA* mutation status and/or HRD status. However, limitations exist with these predictive biomarkers for PARPi response. For example, unexpected discrepancies have been observed between the *BRCA*/HRD status and clinical response to PARPi. Chiang et al. reviewed the concept of synthetic lethality between PARPi and HRD and summarized the findings of the related clinical trials. The authors pointed out that the current *BRCA* gene test and HRD assays are limited by the quality of samples, tumor heterogeneity, and the presence of reversion or secondary mutations of the HR genes. Besides, there is still debate on the appropriate cutoff threshold used in the current HRD assays such as Myriad. The authors also provided a critical discussion on the strengths and weaknesses of some assays in development, including whole exome/genome sequencing-based assays which may be tested in circulating tumor DNA, and functional assays such as RAD51 foci and DNA fiber assays.

DDR involves multiple biological processes such as cell cycle, DNA replication, DNA repair and even immune responses. Enhancer of zeste homolog 2(EZH2) is a catalytic subunit of polycomb repressive complex 2 (PRC2). It is overexpressed in many solid cancers like breast cancer, prostate cancer and EOC. Liu et al. demonstrated that EZH2 is inhibited by microRNAs (miRNAs) such as miR-101-3p, miR-26a-5p and miR-141-3p, and yet EZH2 can also inhibit the miRNA expression by H3K27me3 trimethylation, thus forming a positive feedback loop. This results in persistent elevated expression of EZH2 in EOC, resulting in a reduced expression of tumor-suppressor and cell proliferation-related proteins like p21, p53 and RUNX3 which are important in cell cycle and DNA damage regulation. Three cycles of weekly Intra-tumoral injection of agomir miR-101-3p or agomir miR-26a-5p, where the miRNAs and Argonaute (Ago) proteins form complexes binding to the 3’UTR of EZH2, reduced the tumor volume and the expression of EZH2 and Ki67 in mice inoculated with SKOV3^LV-EZH2^ tumors. The results of this article provided insights on the how EZH2 is regulated *via* miRNAs, and highlights the potential for tumor growth inhibition using EZH2-targeted miRNAs.

DDR is not limited to EOC in gynecologic malignancies. More than 99% of cervical cancer is associated with high-risk human papillomavirus (HPV) infection. HPV enters the basal layer of stratified epithelium through micro-abrasions. Upon entry into the host nucleus, the HPV genome is replicated. The HPV E6 protein degrades p53 and E7 degrades the retinoblastoma suppressor protein pRb, stimulating G1 to S-phase transition and leading to cell proliferation. Radiotherapy can kill cervical cancer cells by inducing DSB. However, HPV-positive cells are more resistant to radiotherapy than HPV-negative cells. Huang et al. showed that Ras-associated binding protein (Rab)12, which regulates transport of circulating endosomes to lysosomal carriers and indirectly stimulates autophagy, had higher expression in HPV-positive cervical cancer cell lines than HPV-negative cell lines. It was up-regulated by HPV E6 and E7, and its mRNA and protein expression was also increased by radiotherapy promoting G2/M arrest by up-regulation of p-Cdc2(Tyr15) and down-regulation of p-Cdc2(Thr161). Knockdown of Rab12 alleviated G2/M arrest, induced DSB, inhibited HRR, and enhanced radiosensitivity. Hence, Rab12 may be a potential target for cervical cancer especially those which are HPV-positive.

This Research Topic has collected review articles that provided a comprehensive overview on DDR targeting, including the use of PARPi and the relevant biomarker tests in EOC. It also contains original research articles that investigated the other molecular pathways related to DNA damage repair that may provide novel therapeutic approaches for EOC and cervical cancer in the future.

## Author Contributions

All authors in this editorial developed this Research Topic and edited the Research Topic. KT wrote the editorial. All authors contributed to the article and approved the submitted version.

## Conflict of Interest

DSPT reports research support from Astra Zeneca, Karyopharm Therapeutics, Bayer, Roche, and has received personal fees and travel support from AstraZeneca, Novartis, Roche, Merck Serono, MSD, Bayer, Genmab, Takeda, Eisai, GSK and Clovis.

The remaining authors declare that the research was conducted in the absence of any commercial or financial relationships that could be construed as a potential conflict of interest.

## Publisher’s Note

All claims expressed in this article are solely those of the authors and do not necessarily represent those of their affiliated organizations, or those of the publisher, the editors and the reviewers. Any product that may be evaluated in this article, or claim that may be made by its manufacturer, is not guaranteed or endorsed by the publisher.
